# TriGlycerides and high-density lipoprotein cholesterol ratio compared with homeostasis model assessment insulin resistance indexes in screening for metabolic syndrome in the chinese obese children: a cross section study

**DOI:** 10.1186/s12887-015-0456-y

**Published:** 2015-09-28

**Authors:** Jianfeng Liang, Junfen Fu, Youyun Jiang, Guanping Dong, Xiumin Wang, Wei Wu

**Affiliations:** Biostatistics Unit of the Children’s Hospital, Zhejiang University, School of Medicine, Hangzhou, 310003 China; Endocrinology Department of the Children’s Hospital, Zhejiang University, School of Medicine, 57 Zhugan Avenue, Hangzhou, 310003 China

**Keywords:** Child obesity, Metabolic syndrome, Biomarker, TriGlycerides (TG) to High-Density Lipoprotein Cholesterol (HDL-C) ratio, Homeostasis Model Assessment Insulin Resistance (HOMA-IR)

## Abstract

**Background:**

Metabolic Syndrome (MS) is prevalant in China, especially according to the pediatric obesity group. Based on the MS-CHN2012 definition for Chinese children and adolescents the need to explore and establish a convienent MS screening become imminent. This study aims to investigate the optimal cut-off values, compare the accuracy for the (TriGlycerides (TG) to High-Density Lipoprotein Cholesterol (HDL-C)) (TG/HDL-C) ratio and Homeostasis Model Assessment Insulin Resistance (HOMA-IR) indexs to identify Metabolic Syndrome in obese pediatric population in China.

**Method:**

A total sample of 976 children (female286 male690, BMI > =95percentile) aged from 6–16 years underwent a medical assessment including a physical examination and investigations of total cholesterol, high-density lipoprotein, low-density lipoprotein, triglycerides, insulin, glucose, and oral glucose tolerance test to identify the components of Metabolic Syndrome. The validity and accuracy between TG/HDL-C ratio and HOMA-IR were compared by Receiver Operating Characteristics analysis (ROC).

**Result:**

TG/HDL-C ratio achieved a larger ROC Area under Curve (AUC = 0.843) than HOMA-IR indexes (0.640, 0.625 for HOMA1-IR, HOMA2-IR respectively) to screen for Metabolic Syndrome. The cut-off values for MS were: TG/HDL-C ratio > 1.25 (sensitivity: 80 %; specificity: 75 %), HOMA1-IR > 4.59 (sensitivity: 58.7 %; specificity: 65.5 %) and HOMA2-IR > 2.76 (sensitivity: 53.2 %; specificity: 69.5 %). The results kept robust after stratified by gender, age group and pubertal stage.

**Discussion:**

TG/HDL-C ratio was a better indicator than the HOMA-IR to screen for a positive diagnosis for MS. Furthermore, the TG/HDL-C ratio was superior to the HOMA-IR indexes even after the control of possible confusions from the gender, age group and puberty stage.

**Conclusion:**

TG/HDL-C ratio proved a better index than HOMA-IR in screening for MS in obese children and adolescents. TG/HDL-C ratio has a discriminatory power in detecting potential MS in the Chinese obese pediatric population.

## Background

The prevalence of obesity has increased dramatically in children and adolescents as China is gradually taking its place as one of the world’s economic giants, it is becoming an important public health problem [1–4]. Metabolic Syndrome (MS) is not rare in children and adolescents. Chinese national nutrition and health survey showed that in the year 2002, the prevalence of MS was 35.2 % in obese children. Ninety-six percent of obese children screened positive for one MS component anomaly, and 74.1 % of obese children had 2 or more abnormal components [5]. Therefore, it becomes imminent to explore an accessible and effective tool to screen obese children for Metabolic Syndrome components.

For a long period anthropometric measurements had been recognized as the convenient indicators in the predicting MS [6–10]. Afterwards Homeostasis Model Assessment Insulin Resistance (HOMA-IR) indexes have been advocated for a close relationship with the components of MS [11–13]. Nevertheless, controversy exists over the variety of indexes used when screening for MS [8, 9, 14]. Recently, a new index called the TriGlycerides (TG) to High-Density Lipoprotein Cholesterol (HDL-C) ratio (TG/HDL-C ratio) has been gaining popularity because of its ability to explain the significant association with insulin resistance or cardiovascular risk factors in adults [15–20] and in children [21–23]. To our knowledge, few studies have been investigated regarding the cutoffs between TG/HDL-C ratio and MS during the childhood [24, 25] . The aim of our study was to investigate the optimal cutoffs of TG/HDL-C ratio, HOMA-IR and compare their accuracy to identify the MS in Chinese obese children.

## Methods

### Study population

This was a cross-sectional study. Study quality was assessed according to the checklist of STARD (STAndards for the Reporting of Diagnostic accuracy studies). 1 069 Obese children and adolescents between 6 and 16 years old, of both genders (female 443, male 626), consecutively registered at the inpatient ward from our clinic, the Children’s Hospital of Zhejiang University School of Medicine– Hangzhou, in China, between May 2007 and June 2013, were invited to participate in the study. A total of 976 (female286 male690) obese schoolchildren with complete record were eligibly included in the current study. the Age- and sex-specific Body Mass Index (BMI) percentiles, developed by the Working Group for Obesity in China, were used to classify participants as obese (BMI ≥ 95 %) [26]. The exclusion criteria were as follows: the known presence of diabetes or high blood pressure, the use of drugs which influence glucose or lipid metabolism (glucocorticoid), specific causes of endocrine or genetic obesity, low birth weight, distress during blood sampling or a difficult phlebotomy (more than 5 min) as well as menstrual cycle changes that indicate the presence of Polycystic Ovary Syndrome in female participants. Signed informed consent was obtained from participants and or parents or guardians. The study was approved by the Research Ethics Committee of the children’s hospital of Zhejiang University School of Medicine. The MS definition in age group was chosen by the MS-CHN2012 definition [27, 28] for all ages by The Chinese Medical Association in 2012 [29].

### Clinical and anthropometric measurements

Subjects’ height and weight were measured according to our standard protocol [30]. BMI was calculated as weight (kg) divided by height squared (m2). Waist Circumference (WC) was measured midway between the lowest rib and the top of the iliac crest. The mean of two measurements made at the end of a normal expiration was used in the analyses. Two measurements of right arm systolic and diastolic blood pressure (SBP and DBP) were performed three times 10 min apart and the mean values of the latter two measurements were recorded. Pubertal development was assessed by Tanner stage of breast development in girls and testicular volume in boys. This assessment was performed visually by two pediatricians of the same gender as the child.

### Laboratory assays

Venous blood samples were collected after an overnight (≥12 h) fast. Subjects also underwent an oral glucose tolerance test (OGTT; 1.75 g of glucose solution per kg, maximum 75 g). The samples were centrifuged, aliquoted and immediately frozen for future analysis in blind of the clinical information. Blood samples were also analyzed for concentrations of plasma glucose, triglycerides (TG), total cholesterol (TC), high-density lipoprotein cholesterol (HDL-C), low-density lipoprotein cholesterol (LDL-C) and insulin. Serum lipids (enzymatic methods) and plasma glucose (glucose oxidase method) were assayed using the Modular DPP automatic biochemistry analysis system (Roche, Rotkreuz, Switzerland). HDL-C and LDL-C were measured directly. Insulin was determined by chemiluminescent micro particle immunoassay (Abbott Park, IL 60064 UK), developed in the key Laboratory at the Children’s Hospital which had an inter-assay coefficients of variation of <9.0 % and no cross-reactivity to proinsulin (<0.05 %).

### Definitions of MS and HOMA-IR calculation

In this study the presence of pediatric Metabolic Syndrome (MS) was determined according to the MS-CHN2012 MS definition [28] for the > =10 years of age group. a diagnosis of MS was made as the presence of abdominal obesity (WC ≥ 90th percentile for age and gender) plus the presence of two or more of the following components: elevated TG (≥1.47 mmol/L), low HDL-C (<1.03 mmol/L), high blood pressure (systolic ≥130 mmHg or diastolic ≥85 mmHg), and elevated blood glucose (≥5.6 mmol/L). For the <10 years of age group the MS definition by the Society of Pediatrics, Chinese Medical Association in 2012 (MS-CHN2012) [29, 31] was used where elevated blood glucose includes impaired fasting glucose and impaired glucose tolerance according to American Diabetes Association classifications [32] as fasting plasma glucose of ≥5.6 to 6.9 nmol/l, and as 2-h post-OGTT glucose of ≥7.8 to 11.0 nmol/l respectively, finally the family hsitory of metabolic syndrome, type 2 diabetes mellitus, dyslipidaemia, cardiovascular disease, hypertension was investigated. Insulin resistance index was calculated by homeostasis model assessment of insulin resistance (HOMA1-IR) as (fasting insulin mU/L) × (fasting glucose mmol/L)/22.5 [33] and the HOMA2-IR index was obtained by the program HOMA Calculator v2.2.2 at http://www.dtu.ox.ac.uk/homacalculator/index.php.

### Statistical analysis

Data was reported as median (interquartile range), and comparisons were performed using Mann–Whitney *U* test. A sample of 26 from the MS group and 26 from the Non-MS group achieved 90 % power to detect a difference of 0.2 between a diagnostic test with a Receiver Operating Characteristic (ROC) Area Under the Curve (AUC) of 0.8, and alterative diagnostic test with an AUC of 0.6 using a two-sided Z-test at a significance level of 0.05, The correlation between the two diagnostic tests is assumed to be 0.6. Prevalence of individual metabolic abnormalities of different groups was compared using the Chi-square test or Fisher’s exact test as appropriate. A receiver operating characteristic (ROC) curve was generated for the total studied population. The areas under the ROC curve (AUC) were calculated to evaluate the accuracy of the indicators by nonparametric method. The greater the AUC, the greater the discriminatory power of them for MS. The optimal cut-off value was denoted by the value that had the acceptable sensitivity, specificity and the closest point to the upper left corner of the ROC curve, which is often selected as the best combination of true-positive rate and false-positive rate [34]. The Z statistic pairwise comparison was used to compare the AUC. Statistical programs available in SAS for Windows (SAS Release 9.2 Cary, NC, USA) were used in this analysis, *P* < 0.05 was defined significance.

## Results

### Clinical Characteristics and metabolic phenotypes of all sample

1 069 Obese children and adolescents of both genders (female 443, male 626) aged from 6-16years were registered in this study. A total of 42 subjects were excluded because they did not satisfy inclusion criteria (31 with difficult blood sampling, 11 with a low birth weight). Other exclusions were twelve subjects diagnosed with early-onset type 2 diabetes mellitus, nine with distress during BP monitoring, twenty with missing data in clinical or laboratory records and ten who refused to participate. Finally 976 participants were included in the analysis datasets. According to the MS diagnosis, overall our study showed that around 25.8 % of the 976 children and adolescents analyzed presented the syndrome, which was more prevalent in larger than 10-year-age obese individuals, especially those at puberty stage. But no difference was found between genders (Table 1).

**Table 1:** Prevalence of the 976 obese children for MS

strata	Non-MS		MS		Total
Sex					
FEMALE	213	74.5 %	73	25.5 %	286
MALE	511	74.1 %	179	25.9 %	690
Age group*					
<10 years	281	80.5 %	68	19.5 %	349
> = 10 years	443	70.7 %	184	29.3 %	627
Pubertal stage*					
Pre-pubertal	372	81.2 %	86	18.8 %	458
Pubertal	352	68.0 %	166	32.0 %	518

*MS* Metabolic Syndrome, *Comparison by Chi-square *P* < 0.05

The basic characteristics of the MS and Non-MS in the children and adolescents that were eligible for this investigation are stratified by the sex, age group and pubertal stage. The MS group individals were elder, had higher BMI than the Non-MS group. The lipid profile can be seen in Table 2. An atherogenic profile was noticed in the MS group with higher LDL-C, lower HDL-C, higher TG, higher HOMA-IR, and higher TG/HDL-C values and the differences were found significant between MS and non-MS groups. For the HOMA-IR and the TG/HDL-C, statistical significant can also be found among the sex, age strata and pubertal stage groups (Table 3).

**Table 2:** Summary characteristics for clinical and metabolic variables categorized by the status of MS

	Non-MS(*n* = 724)			MS(*n* = 252)		
	Median	P 25	P75	Median	P25	P75
Age, years	10.5	8.83	12	11.62	9.79	12.83
BMI, kg/m2	27.3	25.01	29.9	28.92	26.39	32.24
Systolic blood pressure, mmHg	114	105	122	126	112	135
Diastolic blood pressure, mmHg	67	61	72	70	65	78
Triglyceride, mg/dL	1.09	0.84	1.46	1.8	1.34	2.22
HDL cholesterol, mg/dL	1.24	1.1	1.43	0.96	0.85	1.06
TG/HDL-C	0.9	0.63	1.26	1.85	1.34	2.42
LDL cholesterol, mg/dL	2.34	2	2.74	2.55	2.21	3.03
Fasting plasma glucose, mg/dL	5.2	4.8	5.4	5.5	5	5.8
Fasting plasma insulin, mU/L	16.3	10.7	23.7	22	13.2	31.6
HOMA1-IR	3.67	2.39	5.48	5.42	3.23	7.58
HOMA2-IR	2.1	1.4	3.01	2.86	1.73	4.02

*MS* Metabolic Syndrome, *BMI* body mass index, *TG* triglyceride, *HDL-C* HDL-cholesterol, *LDL-C* LDL-cholesterol, *P25* percentile 25, *P75* percentile 75, statistical significance were found in all the variables between the MS and Non-MS by Mann–Whitney test *p* < 0.05

**Table 3:** HOMA-IR and TG/HDL-C categorized by sex, age group and pubertal stage

strata		HOMA1-IR*				HOMA2-IR*				TG/HDL-C*			
		N	Median	P25	P75	N	Median	P25	P75	N	Median	P25	P75
Sex	female	286	4.22	2.66	6.94	286	2.33	1.52	3.64	286	1.08	.72	1.73
	male	690	3.90	2.47	5.87	690	2.17	1.44	3.16	690	1.05	.67	1.58
Age group	<10 years	349	3.20	2.11	4.64	349	1.76	1.18	2.52	349	.95	.65	1.49
	> = 10 years	627	4.55	2.96	7.00	627	2.50	1.70	3.73	627	1.13	.71	1.68
pubertal stage	Pre-pubertal	458	3.56	2.26	5.05	458	2.01	1.27	2.81	458	.94	.64	1.46
	Pubertal	518	4.62	2.87	7.27	518	2.55	1.62	3.88	518	1.18	.76	1.77

*P25* percentile 25, *P75* percentile 75, *statistical significance were found between strata by Mann–Whitney test *p* < 0.05

### Receiver operating characteristics analyses

The TG/HDL-C ratio was a better predictor of MS (acceptable sensitivity and specificity and higher AUC-ROC) than either HOMA1-IR or HOMA2-IR. The cut-off values for MS were: TG/HDL-C ratio > 1.25 (sensitivity: 80 %; specificity: 75 %), HOMA1-IR > 4.59 (sensitivity: 58.7 %; specificity: 65.5 %) and HOMA2-IR > 2.76 (sensitivity: 53.2 %; specificity: 69.5 %). After stratified by age group, puberty stage and sex, the cutoffs of HOMA1-IR changed from 3.58–5.74 while the cutoffs of HOMA2-IR fluctuated from 1.92–2.99. However the cutoffs of TG/HDL-C varied slightly from 1.21–1.53. The Overall AUC-ROC values for the prediction of MS were 0.640, 0.625, and 0.843 by HOMA1-IR, HOMA2-IR and TG/HDL-C respectively. Significant difference of the AUC-ROC values between HOMA-IR and TG/HDL-C was found with a higher sensitivity and specificity. When stratified by age group, gender and puberty stage the AUC-ROC values for the prediction by HOMA-IR were still lower than those by TG/HDL-C.

**Fig. 1:**
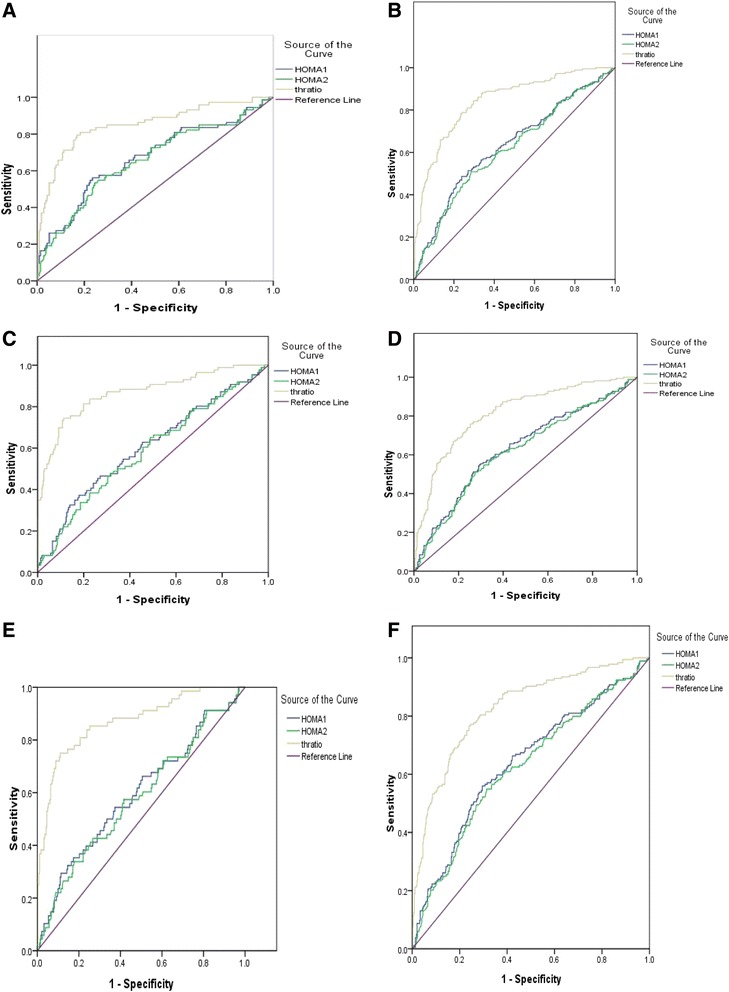
ROC comparisons of HOMA-IR and TG/HDL-C stratified by sex, age group and pubertal stage. AUC-ROC Z statistic for pairwise comparison of AUC: HOMA1-IR = HOMA2-IR, *p* > 0.05; HOMA1-IR < TG/HDL-C, *p* < 0.05; HOMA2-IR < TG/HDL-C, *p* < 0.05; When stratified by sex **a** female, **b** male; age group **c** (<10 years), **d** (> = 10 years); pubertal stage (pre-pubertal stage) (**e**), (pubertal stage) (**f**)

Figure 1 represents the age group, pubertal stage and sex-specific ROC curve analyses, respectively. The ROC curves visually represent the relationship between sensitivity (true positive rate) and 1-specificity (false positive rate) over the entire range of the index value. All the curves (Fig. 1) were significantly greater than what were expected by chance stratification by the age group, puberty stage and sex. Analysis of the data indicated significant differences in ROC curves, with TG/HDL-C performing reasonably better than HOMA1-IR or HOMA2-IR in identifying MS in obese adolescents, and no difference in ROC curves were found between HOMA1-IR and HOMA2-IR (*p* > 0.05).

## Discussion

The present study investigated the optimal cut-off values for TG/HDL-C ratio, and HOMA-IR indexes to identify MS in a pediatric obese population. It was also demonstrated that the TG/HDL-C ratio was a better indicator than the HOMA-IR to screen for a positive diagnosis for MS. Furthermore, it was verified that the TG/HDL-C ratio was superior to the HOMA-IR indexes even after the control of possible confusions from the gender, age group and puberty stage.

Previous studies demonstrated that the HOMA-IR indexes were a good indicator in identifying insulin resistance and MS in children [22, 23] and in adults [20]. But the inconvenience was only a specific range of values are acceptable for calculation. In clinical practice, this limitation complicates the management of insulin results outside the limits and a computer is needed to run the program [25]. Newly published papers revealed that TG/HDL-C ratio makes a significant contribution to the components of the MS [21], but no further investigation had been made to comprehensively develop its association with the screening for MS in Chinese pediatric obesity. In our study the AUC-ROC values (higher than0.8) of TG/HDL-C ratio were much more robust than HOMA-IR indexes and were not much influenced by the pubertal stage. These features make the TG/HDL-C ratio indicator outstanding from other indicators for screening for MS in obese pediatric population.

The TG/HDL-C ratio’s optimal cut off value to screen for MS is reasonable in obese children as the definition of the MS is the high amount of abdominal fat plus two of the four components including the Triglyceride and HDL cholesterol. So there is a high probability to be diagnosed as having MS. However, the optimal ratio of Triglyceride and HDL cholesterol can make a significant discrimination [35] to MS, when TG/HDL-C ratio increased, the trend toward smaller HDL size was obvious, which indicated that the maturation of HDL might be impeded and the reverse cholesterol transport might be weakened [35] and this imbalance of the ratio may reveal the complexity of the metabolic processing. The relationship between TG/HDL-C and MS might be different according to the sex, age and race/ethics due to the different components contributions of MS is depondent on the sex, age and race/ethics. In African-American men, the recommended TG/HDL-C threshold is valid, while In African-American women, the failure of the TG/HDL-C ratio to predict insulin resistance occurred probably due to normal TG levels rather than high HDL-C levels and it is more likely that the African-American women with the metabolic syndrome are to have low HDL-C levels than elevated TG levels based on the observation [36]. Another study suggested that the TG/HDL-C ratio was significantly higher in older women than in younger women, while the ratio was comparable in younger and older men [20]. In our study, the difference of TG/HDL-C between sex, age group and pubertal stage had statistical significance, Female and age group of less than 10 years may have a higher cutoff (1.44, 1.53 respectively) than the overall cutoff. We found a cutoff at 1.25 with a sensitivity of 80 % and specificity of 75 % for TG/HDL-C to screen for MS in Chinese obese children. However, further longitudinal study should be performed to confirm if TG/HDL-C has the advantages [23] of not being age-specific, sex, and is independent of pubertal stage in the Chinese children population.

One limitation of our study might be a potential bias caused by inconsistent measurements of Triglyceride and HDL cholesterol, because only data from patients in only one center with obesity and concomitant diseases are included in this study. Other bias with regard to the study population from a cross-sectional study may also have occurred; therefore the result may lack direct causality. Methodological aspects, such as biochemical measurements are more difficult to standardize in several years and the study result was accomplished only in one center, may contribute to the possible bias. However, experienced pediatricians and team staff in cooperation can make sure to comply with standardized procedures in anthropometric parameter measurement, analytical methodology and lab workup, which should make results from different year data comparable.

## Conclusion

This study demonstrates that TG/HDL-C ratio for screening MS may be a better index than HOMA-IR in screening obese children and adolescents with pediatric MS. We suggest that the accessible, effective and methodologically simple assessment of TG/HDL-C ratio might be powerful in detecting the early stage of potential MS in Chinese obese children and adolescents although further longitudinal study is needed to confirm the result.

## Abbreviations

IDF: International Diabetes Federation
MS: Metabolic Syndrome
HOMA-IR: Homeostasis Model Assessment Insulin Resistance
TG: TriGlycerides
HDL-C: High-Density Lipoprotein Cholesterol
BMI: Body Mass Index
WC: Waist Circumference
SBP: Systolic blood pressure
DBP: Diastolic Blood Pressure
OGTT: Oral Glucose Tolerance Test
TC: Total Cholesterol
LDL-C: Low-Density Lipoprotein Cholesterol
ROC: Receiver Operating Characteristic
AUC: Area Under the Curve
